# Application of orange essential oil as an antistaphylococcal agent in a dressing model

**DOI:** 10.1186/1472-6882-12-125

**Published:** 2012-08-16

**Authors:** Arunachalam Muthaiyan, Debabrata Biswas, Philip G Crandall, Brian J Wilkinson, Steven C Ricke

**Affiliations:** 1 Center for Food Safety and Department of Food Science, University of Arkansas, Fayetteville, AR, 72701, USA; 2 Current address: Department of Animal and Avian Sciences, University of Maryland, College Park, MD, 20742, USA; 3 Microbiology Group, Department of Biological Sciences, Illinois State University, Normal, IL, 61790, USA

**Keywords:** MRSA, VISA, *S. aureus*, Antibiotic resistance, Natural antimicrobials, Orange essential oil

## Abstract

**Background:**

*Staphylococcus aureus* is the pathogen most often and prevalently involved in skin and soft tissue infections. In recent decades outbreaks of methicillin-resistant *S. aureus* (MRSA) have created major problems for skin therapy, and burn and wound care units. Topical antimicrobials are most important component of wound infection therapy. Alternative therapies are being sought for treatment of MRSA and one area of interest is the use of essential oils. With the increasing interest in the use and application of natural products, we screened the potential application of terpeneless cold pressed Valencia orange oil (CPV) for topical therapy against MRSA using an *in vitro* dressing model and skin keratinocyte cell culture model.

**Methods:**

The inhibitory effect of CPV was determined by disc diffusion vapor assay for MRSA and vancomycin intermediate-resistant *S. aureus* (VISA) strains. Antistaphylococcal effect of CPV in an *in vitro* dressing model was tested on *S. aureus* inoculated tryptic soya agar plate. Bactericidal effect of CPV on MRSA and VISA infected keratinocyte cells was examined by enumeration of extra- and intra-cellular bacterial cells at different treatment time points. Cytotoxic effects on human skin cells was tested by adding CPV to the keratinocyte (HEK001) cells grown in serum free KSFM media, and observed by phase-contrast microscope.

**Results:**

CPV vapour effectively inhibited the MRSA and VISA strains in both disc diffusion vapour assay and *in vitro* dressing model. Compared to untreated control addition of 0.1% CPV to MRSA infected keratinocyte decreased the viable MRSA cells by 2 log CFU/mL in 1 h and in VISA strain 3 log CFU/mL reduction was observed in 1 h. After 3 h viable *S. aureus* cells were not detected in the 0.2% CPV treatment. Bactericidal concentration of CPV did not show any cytotoxic effect on the human skin keratinocyte cells *in vitro*.

**Conclusions:**

At lower concentration addition of CPV to keratinocytes infected with MRSA and VISA rapidly killed the bacterial cells without causing any toxic effect to the keratinocytes. Therefore, the results of this study warrant further in vivo study to evaluate the potential of CPV as a topical antistaphylococcal agent.

## Background

The highly adaptive *S. aureus* is the causative agent of a wide variety of human infections, ranging from superficial skin infections to deep abscesses and more serious life threatening infections [1]. Since the 1990s, methicillin-resistant *S. aureus* (MRSA) has accounted for an increasing proportion of community associated infections in the U.S. [1,2]. MRSA has become a primary cause of skin and soft tissue infections among persons without extensive exposure to healthcare settings. Data obtained from nationally representative ambulatory care surveys in the U.S. show that the infections associated with skin and soft-tissue increased from 8.6 million in 1997 to 14.2 million in 2005 [3]. Skin and soft tissue infections (SSTIs) involve microbial invasions of the primary host defence barriers epidermis and underlying soft tissues [4,5]. Likewise, patients hospitalized with burn wounds are at increased risk of developing microbial colonization and infection caused by *S. aureus*. Previous studies revealed that *S. aureus* is the most frequently isolated bacterial species among the other pathogens from the burn wounds [6,7].

Generally dressings and topical antimicrobial agents are routinely used to prevent skin and burn infections and also to keep the wound moist to promote healing [6]. Although, a small number of antibiotics are used as prophylactics to prevent wound infection they are not routinely administered to burn patients due to the high cost and the risk of adverse side effects [8]. Since, the constant use of antibiotics select the bacterial populations including *S. aureus* that are resistant to multiple antibiotics alternative therapies and new medical practices are very much needed [9-11]. One such approach is the search of biologically active pharmacophores from natural resources and traditional medicines [12-16]. Natural products have been investigated and utilized to alleviate disease since early human history. Before the “synthetic era”, 80% of all medicines were obtained from roots, barks, leaves, flowers, seeds and fruits [17].

Numerous studies have explored the promising novel antimicrobial candidates from plant derived essential oils (EOs). These EOs are particularly interesting since some oils have been used by native groups for curative purposes in the past [18,19]. Many plants EOs have demonstrated for antimicrobial activity against variety of bacterial pathogens [20,21]. One such a prominent example is tea tree oil obtained from the Australian tree *Melaleuca alternifolia.* Tea tree oil has been shown to be active against a wide range of microorganisms including *S. aureus*[20,22]. In previous studies the antimicrobial activities of EOs have also been investigated and their actions against various pathogens, including clinical MRSA isolates, have been demonstrated [23-30].

Fisher et al. reported the effectiveness of citrus EOs and vapours of lemon and the citrus EO components citral, limonene, and linalool against a number of common foodborne pathogens *Listeria monocytenes, S. aureus, Bacillus cereus, E.* coli O157, and *Campylobacter jejuni* both *in vitro* and on food models [31]. In our laboratory we have demonstrated the inhibition of *Salmonella*[32], *Listeria*[33,34]*, Escherichia coli* O157: H7 [35], *Campylobacter*[36], and methicillin resistant *S. aureus*[37] by citrus derived cold pressed Valencia orange oil, terpeneless Valencia orange oil, cold pressed orange terpenes, high purity orange terpenes, d-limonene, and terpenes from orange essence. In our previous study the inhibitory and cell wall lytic effect of 0.1% and 0.2% cold pressed terpeneless Valencia orange oil (CPV) against MRSA and VISA was demonstrated by disc diffusion and agar dilution methods and confirmed by genomic transcriptional profiling and electron microscopy [37]. With the increasing interest in the use and application of natural antimicrobial agents for the therapy in the present study we evaluated the potential of CPV for topical therapy against MRSA by determining the antistaphylococcal effect of CPV in dressing model and *S. aureus* infected keratinocyte cell culture study.

## Methods

### Bacterial strains

Methicillin-susceptible *S. aureus* strain SH1000 [38], methicillin-resistant strains COL [39], 13136 p^-^m^+^[40], and N315 [41], and vancomycin intermediate-resistant strains 13136 p^-^m^+^ V_20_[42], and Mu50 [41] were used in this study. Depending on the experimental condition described in the following sections bacterial strains were grown in either tryptic soya broth (TSB) or tryptic soya agar (TSA) media (Difco Laboratories, Inc. Detroit, MI) and incubated at 37°C for 18 h.

### Orange essential oil

Commercially available terpeneless cold pressed Valencia orange oil was obtained from Firmenich Citrus Center, Safety Harbor, FL, USA. CPV is derived from mechanical extraction of the orange oil which is further concentrated under vacuum [43]. The major components of CPV are Linalool 20.2%, Decanal 18%, Geranial 9.1%, α-Terpineol 5.8%, Valencene 5.2%, Neral 5%, Dodecanal 4.1%, Citronellal 3.9%, and Limonene 0.3% [36]. The most predominant compounds are the alcohol linalool (20.2%) followed by decanal (18%), and geranial (9.1%), the amount of limonene is much lower at 0.3% [36].

### Disc diffusion vapor assay for screening the inhibitory effect of CPV

Disc diffusion vapor assay was carried out by the method described by Goñi et al. [44] and Edwards-Jones et al. [45]. Overnight cultures of the *S. aureus* (7 log CFU/mL) were streaked on sterile TSA (Difco Laboratories, Inc.) using a cotton swab dipped into the culture. The swab was used to streak the agar plate to produce a lawn of growth by streaking the plate in 3 different directions. Ten μL of 100% CPV was aseptically pipetted onto sterile 6-mm paper discs (Becton Dickson, Franklin Lakes, NJ) and subsequently the CPV impregnated paper discs were aseptically placed in the centre of the lid of the Petri dish. Control plates were prepared by adding 10 μL of sterile water to the filter discs. The Petri dishes were subsequently sealed using parafilm and incubated at 37°C in an inverted position. The diameters of zones of inhibitions were measured in mm after 24 h of incubation. The assays were carried out on three different occasions in duplicate.

### *In vitro* dressing model study

**Figure 1:**
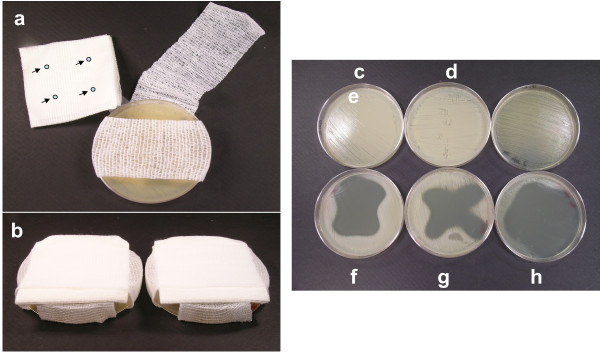
**Dressing model using an agar plate.** (**a**), arrows indicate the CPV spots on gauze dressing pad; (**b**), complete setup of dressing model with gauze dressing pad wrapped with bandage. Untreated control plates of *S. aureus* strains (**c**), COL; (**d**), Mu50; and (**e**), 13136 p^-^m^+^ V_20_. Inhibition of *S. aureus* (f), COL; (g), Mu50; and (h), 13136 p^-^m^+^ V_20_ caused by CPV

*In vitro* dressing model was designed by a modification of the method described by Edwards-Jones et al. [45]. Briefly, 10 μL aliquot of CPV (100%) was spotted in four different areas on the sterile 10.2 x 10.2 cm, 12 ply cotton gauze dressing pad (Duka Corp. Happauge, NY) (Figure 1a). For control plates 10 μL of sterile deionized water was spotted in the gauze dressing pad. TSA plates were seeded with a suspension of 7 log CFU/mL of MRSA strains COL, Mu50 or VISA strain 13136 p^-^m^+^ V_20_ and covered with single layer of sterile bandage (QMD Medical, Quebéc, Canada) without touching the inoculated agar surface. As shown in Figure 1a CPV spotted gauze dressing pad was placed on the bandage by CPV spots facing towards the inoculated agar surface and wrapped with bandage to hold the gauze dressing pad on the Petri plate (Figure 1b). The dressing model Petri plates were subsequently incubated at 37°C for 24 h and the treated plates were compared with untreated control to identify the visible inhibition zones. This study was carried out on three different occasions in duplicate.

### Keratinocyte cell culture

*Homo sapiens* skin keratinocyte (HEK001) ATCC CRL-2404™ cells were grown in keratinocyte serum free media (K-SFM) with two additives, bovine pituitary extracts (BPE) and human recombinant epidermal growth factor (EGF) (Invitrogen, Carlsbad, CA). Cells were grown routinely in a 75 cm^2^ flask at 37°C in a 5% CO_2_-humidified incubator. Ninety percent confluent cultures were trypsinized, and new cultures were prepared by seeding with 10^5^ cells/ml in 75 cm^2^ flasks. For cytotoxicity and infection assays, BD Falcon™ 24-well tissue culture plates (BD, Franklin Lakes, NJ) were seeded with 10^5^ cells/ml/well and incubated at 37°C in a humidified 5% CO_2_ incubator for 18 to 20 h to obtain a semi-confluent monolayer. Prior to assays, the monolayers were washed and incubated with K-SFM medium without antibiotic.

### Cytotoxicity assay

The MIC of CPV for the 6 *S. aureus* strains used in this study was previously determined as 0.18% for the strains 13136 p^-^m^+^ and 13136 p^-^m^+^V_20_ and 0.2% for strains COL, Mu50, and N315 [37]. Based on our previous results, 0.1% and 0.2% of CPV was prepared using dimethyl sulfoxide (DMSO) as a dispersing agent and added to the HEK001 cell monolayers and incubated for 24 h. After incubation, the cell monolayer was observed under phase-contrast microscope (Olympus, Japan). One set of HEK001 cell monolayer without any treatment and another set with equal volume of DMSO used in the CPV treatment were used as controls. To compare a typical cytotoxicity one set of HEK001 cell monolayer was treated with a known skin irritant SDS (25 μg/mL) [46].

### *S. aureus* infection assay

Adherence and invasion assays were performed using a modified procedure derived from Harvey et al. [47]. Briefly, one loopful of overnight grown bacterial cells were collected from TSA plates and suspended in K-SFM media with additives. Aliquots (100 μl) of the bacterial suspension, containing approximately 7 log CFU/mL [multiplicity of infection (MOI) 1:100], were inoculated in duplicate in a 24-well tissue culture plate containing semi-confluent HEK001 cell monolayers. The CFUs of bacteria were determined simultaneously by serial dilution plate count on TSA plates. Infected monolayers were incubated for 2 h at 37°C under a 5% CO_2_ humidified atmosphere to allow the bacterial cells to adhere and infect the HEK001 cells. Infected HEK001 monolayers were washed five times with K-SFM medium and re-incubated for 3 h in fresh media containing 0.1 or 0.2% CPV. For controls, infected HEK001 monolayers were re-incubated with fresh K-SFM medium alone or K-SFM medium with equal volume of DMSO used in CPV treatment. After 1, 2, and 3 h of incubation both controls and CPV treated HEK001 cells were lysed with 0.1% Triton X (Sigma, St. Louis, MO) in PBS for 15 min to detach the bacterial cells and subsequently the lysate containing bacterial cells were diluted and viable intra- and extra-cellular bacterial numbers were determined by counting the CFU on TSA plate. Results are expressed as the average number of bacterial CFU from three assays.

## Results and discussion

### Effect of CPV vapour on *S. aureus*

In an effort to explore the potential use of orange EO against antibiotic resistant *S. aureus,* in our earlier study inhibitory effects and mode of action of CPV against methicillin-susceptible strain SH1000, MRSA strains COL, 13136 p^-^m^+^, and N315, and VISA strains 13136 p^-^m^+^ V_20_ , and Mu50 were studied [37]. Compared to other tested EOs CPV effectively inhibited all the tested *S. aureus* strains [37]. Therefore, in the present study the effect of CPV vapour on *S. aureus* was examined on both agar plates and an *in vitro* dressing model to evaluate the potential of CPV for topical therapy. Generally to study the antimicrobial effect of vapour, EO impregnated paper disc is placed on the lid of Petri dish and subsequently the growth inhibition zone is measured and used to indicate the antimicrobial effect of EO [19,21].

In our disc diffusion CPV vapour study, growth of all the six *S. aureus* strains were inhibited at different levels (Table 1). The maximum diameter of inhibition zone 78.8 ± 1.8 mm was observed in VISA strain 13136 p^-^m^+^V_20_ followed by the MRSA strains 13136 p^-^m^+^, COL, N315, and Mu50. The lower diameter of inhibition zone 17.8 ± 1.4 mm was observed in an antibiotic susceptible strain SH1000. Similar to our observation, strain specific variation in the growth inhibition in *S. aureus* was previously observed by Edwards-Jones et al. [45]. In their study, the most effective combination of geranium and commercial grapefruit extract Citricidal™ vapour against MRSA did not affect the growth of antibiotic susceptible *S. aureus* strain NCTC 6571 [45]. It has been previously noted that the degree of inhibition of bacterial growth by EOs considerably varies due to the complexity of EOs and characteristics of bacterial strains [48]. Related to results observed in our study Gaunt et al., [49] demonstrated the antistaphylococcal effect of evaporated volatile compounds from orange oil. In their study, agar plates inoculated with *S. aureus* strain 8532 were exposed in a large air-tight chamber to candle flames combined with the volatile bactericidal compounds *β*-pinene and orange oil and results showed that compared to plain candle addition of volatile oils significantly reduced the number of *S. aureus* colonies. Goñi et al. [44] also reported the susceptibility of various strains of Gram-negative and -positive microorganisms including *S. aureus* to vapour phase cinnamon, clove and a mixture of cinnamon and clove EOs. In their study they found that vapour phase of cinnamon, clove or mixture of cinnamon and clove EOs showed better inhibition of *S. aureus* growth than the direct contact disc diffusion method [44].

**Table 1:** **Inhibitory effect of terpeneless cold pressed Valencia orange oil (CPV) against *S. aureus *strains determined by a disc-diffusion vapor assay**

***S. aureus *strain**	**Inhibition Zone (mm)**^**a**^
SH1000	17.8 ± 1.4
COL	64.0 ± 2.2
13136 p^-^m^+^	70.2 ± 1.4
13136 p^-^m^+^V_20_	78.8 ± 1.8
N315	62.6 ± 2.5
Mu50	21.0 ± 2.6

^a^ Inhibition zones are average values of three independent trials Â± standard deviation of the mean (SD, n=6).

### Antistaphylococcal effect of CPV vapor in dressing model

Our disc diffusion vapor assay proved that the vapor phase of the volatile components in the CPV serves as possible source of antimicrobial agents. Therefore, we considered assessment of the anti-staphylococcal effect of CPV vapor in an *in vitro* dressing model to evaluate the potential topical application of CPV. In this experiment the inhibitory effect of CPV was qualitatively evaluated by the presence or absence of inhibition zone by comparing the test and control dressing models (Figure 1c-h). As we observed in the disc diffusion vapor assay a similar inhibitory effect was observed in the dressing model as well. Growth of MRSA strains COL and Mu50 and VISA strain 13136 p^-^m^+^ V_20_ was inhibited by the vapor released from the CPV spotted gauze dressing pad and exhibited a clear inhibition zones on agar plates (Figure 1f-h). Our present study has demonstrated the potential of the CPV as an anti-MRSA agent in the vapor phase in both the disc diffusion assay and the *in vitro* dressing model. Effectiveness of the vapor phase EOs against microbial growth was shown to be better than the direct contact of EOs with the inoculated culture has already been reported in other studies [44,50]. In an *in vitro* dressing model study Edwards-Jones et al. [45] demonstrated the inhibitory effect of combination of geranium and commercial grapefruit extract Citricidal™ vapor against MRSA. Results of their study concluded that EOs can be applied as a natural anti-MRSA agent on the outer layer of the dressing without disturbing the normal wound healing process.

### Non-cytotoxic effect of CPV on HEK001 keratinocyte cells

**Figure 2:**
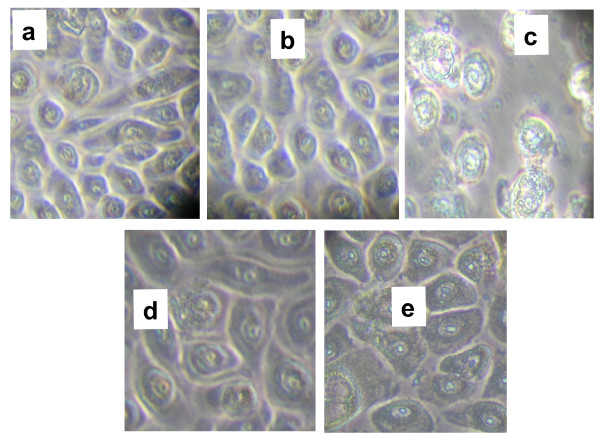
**Effect of CPV in *Homo sapiens *skin keratinocyte (HEK001) ATCC** CRL-2404™ **cell monolayer.** (**a**), normal HEK001 keratinocyte cell monolayer; (**b**), HEK001 keratinocyte cell monolayer treated with equal volume of DMSO used in CPV treatment; (**c**), HEK001 keratinocyte cell monolayer exhibiting cytotoxic effect caused by SDS (25 μg/mL); (**d**), HEK001 keratinocyte cell monolayer treated with 0.1% CPV; (**e**), HEK001 keratinocyte cell monolayer treated with 0.2% CPV. Magnification , x400

Dermal exposure to either synthetic or natural chemical substances can lead to a wide variety of skin reactions. Therefore, it is important to evaluate the new topical therapy agents that can potentially affect the skin cells [51]. Skin is the first-line defense against invading pathogens [52], and is composed of three layers, whereas the outermost epidermis is a squamous epithelium that mainly consists of keratinocytes [53]. Therefore, in this study we used keratinocyte cells as an *in vitro* model to assess the effect of CPV. Since there are general concerns about the toxicity and adverse effects of the plant derived natural products on the host first we evaluated the toxic effect of CPV on human keratinocyte cell monolayer. In our previous study we have shown that the MIC of CPV for the *S. aureus* strains used in the present study as 0.18% for the strains 13136 p^-^m^+^ and 13136 p^-^m^+^V_20_ and 0.2% for strains COL, Mu50, and N315 [37]. Therefore, to examine whether CPV has toxic effect on keratinocyte cells, 0.1% (½ x MIC) and 0.2% (1x MIC ) of CPV was added to the HEK001 keratinocyte cell monolayer and subsequently at regular intervals the cell monolayers were examined under phase-contrast microscope for any morphological changes caused by the toxic effect of CPV. Figure 2a is a representative picture of normal keratinocyte cells and DMSO treated cells did not show any toxic effect and looks similar to normal keratinocyte cells (Figure 2b). Figure 2c represents the cytotoxic effect caused by a known skin irritant SDS on cell monolayer. Compared to controls (Figure 2a-c), CPV (0.1% and 0.2%) treated keratinocyte monolayer cells did not exhibit any toxic response even after 24 h of incubation (Figure 2d,e). Result of this observation indicated that the concentration (0.1 and 0.2%) of CPV that has previously shown to be bactericidal for MRSA and VISA cells did not produce any adverse effect on human epidermal keratinocyte cells [37]. Host tolerance is one of the issues that must be considered when evaluating natural antibacterial agents [54]. Many currently available antifungal and antibacterial agent possess undesirable toxicity [16,54]. However, interestingly in our *in vitro* study the CPV treatment on keratinocyte cells did not exhibit any toxic effect mediated cell death in keratinocyte cells. It indicates the potential suitability of CPV for the topical antimicrobial treatment for dermal *S. aureus* infections.

### Anti-staphylococcal effect of CPV in *S. aureus* infected keratinocyte cells

Enumeration of total number *S. aureus* cells infected in HEK001 keratinocyte monolayer indicated that addition of CPV (0.1% or 0.2%) into the infected cells reduced the adhesion of MRSA strain COL and VISA strain 13136p^-^m^+^V_20_ to the keratinocyte cells. Infected HEK001 monolayers treated with fresh K-SFM medium alone or K-SFM medium mixed with equal volume of DMSO used in CPV treatment were used as controls in this experiment. We did not observe any difference between these two controls (data not shown). Compared to the control, CPV treated keratinocyte exhibited gradual decreases in total numbers of attached *S. aureus* cells within 3 h of treatment. After 3 h of incubation with 0.2% CPV number of viable COL and 13136p^-^m^+^V_20_ cells became undetectable (Table 2). There are also several clinical studies [55,56] and case reports [57,58] reporting the successful use of EOs in treating MRSA nasal carriage or MRSA infections. Dryden *et al*. [55] and Caelli *et al*. [59] reported that a topical tea tree oil treatment was as effective as standard therapy for reducing MRSA nasal colonization. Sherry *et al*. demonstrated the successful use of the commercial phytochemical mixture Polytoxinol™ containing the extracts from Lemongrass, *Eucalyptus, Melaleuca,* Clove, Thyme as well as B.H.T. (Butylated Hydroxy Toluene), Triclosan (0.3%) and 95 undematured ethano (69.7%) to treat an intractable MRSA infection of the tibia in an adult patient [58]. Previous case studies have also demonstrated the topical use of *Eucalyptus* oil extracted from the *Eucalyptus globulus* leaf together with bioethanol for MRSA wound infection. This case study has reported that without administering any antibiotic application of 0.5 g of the oil per day to the wound for three weeks improved wound healing with clearing the MRSA to an undetectable level [60]. Recently, Palaniappan and Holley showed that natural antimicrobials carvacrol, thymol, and cinnamaldehyde were able to substantially decrease the MIC of antibiotics in a diverse group of bacteria containing genetic elements responsible for drug resistance [61]. They have demonstrated the synergistic effect of carvacrol, thymol, and cinnamaldehyde in the reduced MIC's of ampicillin, penicillin and bacitracin against penicillin-resistant *S. aureus*[61]. Thus, we speculate it is possible to use the CPV either single topical agent or synergistically with other antibiotics to control the *S. aureus* skin infections.

**Table 2:** **Effect of CPV treatment on the total number of *S. aureus *infected with skin keratinocyte (HEK001) cell monolayer**

***S. aureus *Strain**	**Initial Inoculum**^**a**^**(Log CFU/mL)**	**Incubation**	**Control**	**CPV Treatment**	
		**Time (h)**	^**a**^**(Log CFU/mL)**	^**a**^**(Log CFU/mL)**	
				**0.1%**	**0.2%**
COL	7.48 ± 0.16	1	5.53 ± 0.45	2.84 ± 0.20	2.18 ± 0.42
		2	5.79 ± 0.07	2.06 ± 0.38	1.60 ± 0.00
		3	6.30 ± 0.07	1.98 ± 0.45	ND
13136 p^-^m^+^ V_20_	7.34 ± 0.11	1	5.04 ± 0.13	3.47 ± 0.20	2.62 ± 0.05
		2	5.58 ± 0.06	2.83 ± 0.15	1.68 ± 0.15
		3	5.88 ± 0.07	2.29 ± 0.62	ND

^a^ Inhibition zones are average values of three independent trials Â± standard deviation of the mean (SD, n=6).

ND – Not detectable.

## Conclusions

MRSA is common in the U.S. and it accounts for more than half of all soft-tissue and skin infections [2]. Surveillance reports indicate that in the U.S. annual MRSA prevalence continuously increased over the 10-year period from 32.7% in 1998 to 53.8% in 2007 and during the 1999–2006 the percentage of *S. aureus* infections resistant to methicillin increased greater than 90% in outpatients admitted to U.S. hospitals. Also, MRSA-related hospitalization rate per 1,000 discharges doubled in 2007 [62,63]. Despite major advances in wound and burn management in the new millennium, infection still remains an important factor in wound healing [6]. Novel classes are clearly needed for MRSA, because current drug classes exhibit emerging resistance. We have initiated this study as a first step towards the investigation of potential antistaphylococcal effect of CPV and its usage for skin infection. In this study, we have demonstrated the antistaphylococcal effect of CPV in an *in vitro* dressing model and *S. aureus* infected keratinocyte cell culture study. Overall in the present study our findings suggest that further *in vivo* studies of CPV are warranted since the CPV showed the inhibition and bactericidal effect on MRSA and VISA in the *in vitro* models. While, we provide interesting and valuable basic data for CPV we suggest prior to continuing on for further studies to test the clinical safety and efficacy of the CPV to use as topical anti-MRSA agent comparison of the inhibitory effect of CPV with antibiotics currently used to treat MRSA skin infection would provide additional valid information for the future therapeutic applications.

In our earlier study we have reported the bacteriolytic effect and mechanism of action of CPV in antibiotic resistant *S. aureus*[37]. The next step in this CPV series study focuses the comparison of the inhibitory effect of CPV with standard antibiotic disc assay together with a quality control strains to develop a standard evaluation method to test the inhibitory effect of CPV on more *S. aureus* strains and other pathogens.

## Abbreviations

CPV: Terpeneless cold-pressed valencia orange oil; EO: Essential oil; MRSA: Methicillin-resistant *Staphylococcus aureus*; VISA: Vancomycin intermediate-resistant *Staphylococcus aureus*.

## Competing interests

The authors declare that they have no competing interests.

## Authors' contributions

AM designed and performed the experiments, interpreted the results and drafted the manuscript. AM and DB designed and performed the cell culture experiment and data analysis. PGC, SCR, and BJW participated in the design of the study. SCR critically read the manuscript and participated in revision of the manuscript. All authors have read and approved the final manuscript.

## Pre-publication history

The pre-publication history for this paper can be accessed here:

http://www.biomedcentral.com/1472-6882/12/125/prepub
